# Persistence of Hepatitis A Virus RNA in Water, on Non-porous Surfaces, and on Blueberries

**DOI:** 10.3389/fmicb.2021.618352

**Published:** 2021-02-04

**Authors:** Mathilde Trudel-Ferland, Eric Jubinville, Julie Jean

**Affiliations:** Institute of Nutrition and Functional Foods (INAF), Université Laval, Quebec City, QC, Canada

**Keywords:** persistence, RNA, hepatitis A, water, food contact surfaces, blueberries

## Abstract

Enteric viruses, such as human norovirus and hepatitis A virus (HAV), are the leading cause of transmissible foodborne illness. Fresh produce such as berries are often contaminated by infected food handlers, soiled water, or food contact surfaces. The gold-standard method for virus detection throughout the food chain is RT-qPCR, which detects portions of genomes including non-infectious viral particles and naked viral RNA. The aim of this study was to evaluate the persistence of heat-inactivated HAV in water, phosphate-buffered saline, on stainless steel and polyvinyl chloride, and on blueberries at −80°C, −20°C, 4°C, and room temperature. In water and phosphate-buffered saline, viral RNA could be detected for up to 90 days regardless of temperature when the initial load was 2.5 × 10^4^ or 2.5 × 10^6^ genome copies. It was detected on polyvinyl chloride and blueberries under most conditions. On stainless steel, the large initial load persisted for 90 days, while the medium-level load was detected only up to 16 days at room temperature or 60 days at 4°C. The detection of non-infectious viral RNA can confound investigations of gastroenteritis outbreaks. Pretreatments that discriminate between naked RNA, non-infectious virions and infectious virions need to be included in the RT-qPCR method in order to reduce the risk of positive results associated with non-infectious viral particles.

## Introduction

Enteric viruses such as human noroviruses (NoV) and hepatitis A virus (HAV) are a leading cause of foodborne illness worldwide. During the past decade, NoV infections numbered at least 124 million per year and HAV infection numbered 13 million per year (WHO, 2015). NoV usually leads to acute gastroenteritis after an incubation period of 24–48 h (Patel et al., 2009). Although the illness lasts for only 2 or 3 days in most cases, its prevalence makes it a global economic burden (Patel et al., 2009; Bartsch et al., 2016a). In the case of HAV, the incubation period ranges from 14 to 28 days, and the subsequent acute hepatitis may last up to 2 months (Lemon et al., 2018). Asymptomatic infections are fairly common, and the disease is endemic in many countries, the majority of cases occurring in early childhood. Although HAV infection is less common in countries with high standards of hygiene, symptomatic infection with a higher risk of hospitalization and death is more frequent since few adults have acquired immunity (Koopmans and Duizer, 2004; Jacobsen, 2018). As international trade accelerates, non-endemic countries are experiencing increased numbers of outbreaks of foodborne viral diseases (Tavoschi et al., 2015; Jacobsen, 2018).

Enteric viruses are excreted in high concentrations in feces and to a lesser extent in vomitus and aerosols. Infected individuals can excrete anywhere from 10^6^ to a 10^11^ copies of NoV or HAV genome per gram of feces (Tjon et al., 2006; Atmar et al., 2008; Bonifait et al., 2015) and may therefore contaminate ready-to-eat or minimally processed foods such as fruits, vegetables, and shellfish as well as surfaces where these products are sorted and packaged. Other contamination routes are sewage-polluted irrigation water and fishing sites (Kokkinos et al., 2012; Lopman et al., 2012; Verhaelen et al., 2013; Randazzo and Sánchez, 2020). Leafy vegetables, such as lettuce, mixed salads, and green onions, are the most frequently contaminated vegetables (Wheeler et al., 2005; Herman et al., 2015), whereas berries are frequently contaminated fruits (Chatziprodromidou et al., 2018; Nasheri et al., 2019). Enteric viruses can remain infectious under harsh environmental conditions, and imported frozen fruits have been involved in recent outbreaks in industrialized countries (Bernard et al., 2014; Collier et al., 2014; Severi et al., 2015; Franklin et al., 2019; Nasheri et al., 2019). This has led governmental bodies such as the *U.S. Food and Drug Administration* and the *Canadian Food Inspection Agency* to establish surveillance plans for viruses in fresh and frozen fruits (Canadian Food Inspection Agency, 2017; U.S. Food and Drug Administration, 2020a).

For detecting viruses in foods, genomic methods have been preferred over titration of infectious particles because they are less tedious and yield results much sooner (Croci et al., 2008). However, these methods raise the issue of non-infectious viral RNA potentially influencing decision-making. This has been examined in the CFIA surveillance report on viruses in fruits and the difficulty of assessing real health risks associated with the consumption of foods positive for viral RNA (Canadian Food Inspection Agency, 2017). The FDA currently recommends using RT-qPCR to detect HAV or NoV in berries, followed by Sanger sequencing to confirm a positive result before suggesting that the producer or distributor involved recalls the lot (U.S. Food and Drug Administration, 2020a,b). Since such product recalls can be financially ruinous to businesses in the food industry, means of recognizing positive tests that are due only to infectious RNA must be found.

Noroviruses and HAV both have a positive-sense, single-stranded RNA genome enclosed in a non-enveloped capsid, the latter being a key factor in their persistence in the environment and resistance to viral inactivation treatments (Girard et al., 2010; Vasickova et al., 2010; Jean et al., 2011; Escudero et al., 2012; Cook et al., 2018; Leblanc et al., 2019). It is widely accepted that infectious NoV, HAV, and their surrogate viruses persist in different waters (Biziagos et al., 1988; Cook et al., 2018), on common surfaces in the food industry (D’Souza et al., 2006; Mattison et al., 2007; Bae et al., 2014), and in food matrices (Butot et al., 2008; Lamhoujeb et al., 2008; Leblanc et al., 2019). However, data on the persistence and significance of non-infectious viral RNA on or in foods are extremely scarce (Limsawat and Ohgaki, 1997; D’Souza et al., 2006; Escudero et al., 2012).

The aim of this study was to evaluate the persistence of inactivated HAV detectable by RT-qPCR in pure water (molecular biology grade) and phosphate-buffered saline, on common non-porous surfaces (stainless steel and polyvinyl chloride), and on blueberries. The effects of temperature, time, and initial concentration on the stability of the inactivated viral genome were examined.

## Materials and Methods

### Preparation of the Viral Stock Suspension

FRhK-4 cells (ATCC^®^ CRL-1688) were cultured as described previously (Mbithi et al., 1991). Briefly, cells were grown in Eagle’s Minimal Essential Medium (product 320-005-CL, Wisent Inc., St-Bruno-de-Montarville, QC, Canada) supplemented with 10% fetal bovine serum (product 080–150, Wisent Inc., Canada), 2 mM L-glutamine (product 609-065-EL, Wisent Inc., Canada), 1% non-essential amino acid mixture (product 321-011-EL, Wisent Inc., Canada), 10 mM HEPES (product 330-050-EL, Wisent Inc., Canada), 0.113% sodium bicarbonate (product 609-105-EL, Wisent Inc., Canada), 50 IU/mL penicillin, and 50 μg/mL streptomycin (product 450-200-EL, Wisent Inc., Canada).

Cytopathogenic HAV strain HM-175, obtained from the Bureau of Microbial Hazards, Health Canada, Ottawa, ON, was propagated as described previously (Mbithi et al., 1992). Viral titer was measured by plaque assay as described previously (Mbithi et al., 1991). Briefly, FRhK-4 monolayers were grown in 12-well culture plates (product3336, Corning Inc., Glendale, AZ, United States) for 24 h at 37°C with 5% CO_2_. Serial dilutions (300 μL) were inoculated in duplicate. Phosphate-buffered saline (PBS, product 21-040-CV, Corning Inc., United States) alone was included as a negative plaque assay control. The plates were incubated at 37°C for 90 min, oscillated gently every 30 min and then overlaid with 3 mL of Minimum Essential Medium (product 220-005-XK, Wisent Inc., Canada), containing 2% fetal bovine serum, 2 mM L-glutamine, 1% non-essential amino acids, 10 mM HEPES, 0.113% sodium bicarbonate, 50 IU/mL penicillin and 50 μg/mL streptomycin, 0.5% magnesium chloride (product CAJT244-1, Avantor Inc., Radnor, PA, United States), and 1% ultra-pure agarose (product 15510-027, Invitrogen Canada Inc., Burlington, ON, Canada). The plates were incubated for 8 days at 37°C with 5% CO_2_, the overlay was then removed, and the cell layers were fixed and stained as described previously (Sattar et al., 1989). The HAV stock suspension (2.24 × 10^5^ pfu/mL) was stored at −80°C until RNA extraction.

### Inactivated Viral Stock Suspension

Concentrated inactivated HAV was prepared as described elsewhere (Suresh et al., 2019) with modifications. Briefly, the HAV stock suspension was diluted 1/10 in PBS and then inactivated at 100°C for 10 min in a water bath. Inactivation was confirmed by plaque assay. Inactivated HAV was quantitated by RT-qPCR. The stock suspension of inactivated HAV (1.22 × 10^5^ genome copies/μL) was stored at −80°C until experiments.

### Real-Time Reverse-Transcriptase PCR

The HAV genome was amplified using iTaq Universal probe 1-step (Bio-Rad, Hercules, CA, United States) on an ABI7500 real-time PCR System (Applied Biosystems, Thermo Fisher Scientific, Waltham, MA, United States) using the SDSv1.3 program. Primers [Integrated DNA Technologies (IDT), Coralville, IA, United States] and probe (Applied Biosystems, United States) were used as described previously (Costafreda et al., 2006). The reaction volume consisted of 10 μL of iTaq universal probes reaction mixture (2×), 250 nM of each primer and probe, 0.5 μL of iScript advanced reverse transcriptase, 2.63 μL RNAase-free water, and 5 μL of RNA sample for a total reaction volume of 20 μL. All samples were plated manually in 96-well half-skirt clear PCR microplates (product PCR-96-HS-AC-C, Axygen, Glendale, AZ, United States) and then sealed with optical adhesive film (LS4360954, Applied Biosystems, United States). Sample and procedural control reactions were performed in duplicate. Molecular -biology-grade water (product L0201, VWR, United States) was used as a negative RT-qPCR control. Reverse transcription was performed at 50°C for 10 min followed by polymerase activation and DNA denaturation at 95°C for 3 min and 45 cycles of 95°C for 15 s and 60°C for 30 s as recommended by the manufacturer. ROX dye was used as a reference dye. Genome copies were quantified using a dsDNA standard curve generated using sequential 10-fold dilutions of dsDNA plasmid pIDTSmart-AMP (IDT, United States) carrying the target sequence described in ISO 15216-1:2017 Appendix G (Anonymous, 2017). Standard curve reactions were performed in triplicate. All standard curve efficiencies were between 90 and 110% with *R*^2^ values superior to 0.98 and thus met the ISO standard.

### Test Surfaces

#### Non-porous Surfaces

Disks of stainless steel (Acier Inoxydable Den-Mar Inc., Quebec City, QC, Canada) and polyvinyl chloride (PVC type1, grade1, Plastique Polyfab, Quebec City, QC, Canada) 1 cm in diameter were cleaned in 6.5% sodium hypochlorite for 15 min, rinsed in sterile deionized water and then cleaned in 70% ethanol for 15 min and allowed to dry. Stainless steel was then autoclaved at 121°C for 20 min, and PVC was decontaminated on each side using UV (254 nm) for 15 min in a laminar flow hood (Jean et al., 2011). The sterile disks were placed in 12-well plates prior to application of inactivated HAV.

#### Food Surface

Blueberries purchased in a local supermarket were washed three times in sterile deionized water. The fruits were dried, disposed calyx down in 12-well plates, and decontaminated using UV light for 30 min prior to application of inactivated HAV (Girard et al., 2016).

### RNA Persistence Study

Microtubes received inactivated HAV (containing RNA) stock suspension diluted to 2.5 × 10^6^, 2.5 × 10^4^, or 2.5 × 10^2^ genome copies per 100 μL of PBS or molecular-biology-grade water. Negative procedural control microtubes received 100 μL of diluent alone. The microtubes were stored at −80°C, −20°C, 4°C, or 23°C (controlled ambient temperature). Inactivated HAV was assayed after 30 min, 6 h, 24 h, 48 h, 4 days, 8 days, 16 days, 2 months, and 3 months. Frozen solution samples were thawed on ice for the same duration. Furthermore, all procedural controls were submitted to the same conditions. Positive procedural controls (positive control T_0_) consisting of fresh dilutions (in PBS or water) of the inactivated HAV suspension were prepared on the day of analysis and included in each assay. These controls were used to calculate the RNA detection in solutions.

For surfaces, 20 μL of inactivated HAV (containing RNA, capsids, etc.) dilution thereof was placed on a disk or blueberry and allowed to dry for 1 h in a PCR workstation AC600 (AirClean Systems, Creedmoor, NC, United States). Two initial loads were tested: 2.5 × 10^6^ and 2.5 × 10^4^ genome copies per repetition. Negative procedural control surfaces received 20 μL of diluent alone. The end of the drying period marked time0. Disks were stored at 4°C and 23°C, whereas blueberries were stored at 4°C and −20°C. Inactivated HAV was assayed at the same time points as in the persistence experiment in solutions. The blueberry surface was not analyzable beyond 16 days when stored at 4°C. Inactivated HAV was eluted from the surfaces using a modified version of a technique described previously (Escudero et al., 2012). Briefly, 50 μL of molecular-biology-grade water was pipetted 25 times over the surface and then collected. The process was repeated with another 50 μL of water to obtain a final eluate volume of 100 μL. Positive procedural controls consisting of a fresh dilution of inactivated HAV suspension were included at each time point. These controls prepared on the day of analysis allow to calculate the RNA detection on surfaces ([RNA]_*positive control T0*_). Previously described positive controls were also used to measure the inactivated HAV elution efficiency on PVC (45%), SS (27%), and blueberries (21%; data not shown). Eluates were analyzed immediately and then stored at −80°C. RNA detection was calculated for the test samples using the following equation unless written otherwise:

$$\mathrm{RNA}\mathrm{detected}\left(\%\right)=\frac{{\left[\mathrm{RNA}\right]}_{\mathrm{sample}\,T_{n}}}{{\left[\mathrm{RNA}\right]}_{\mathrm{positive}\,\mathrm{control}\,T_{0}}}\times 100$$

where [RNA] is the copy number.

However, presence or absence of genome copies was analyzed in water containing 2.5 × 10^2^ genome copies/100 μL and PBS solutions.

### Statistical Analysis

All RT-qPCR assays were performed in duplicate, and all experiments were replicated three times except for analyses in PBS, which were performed twice. Data were analyzed using GraphPad Prism version 8.4.0. A two-way analysis of variance (ANOVA) followed by a Tukey’s multiple comparisons test was used to determine the temperature × time interaction and the initial inactivated HAV load × time interaction effects on % RNA detected in suspensions. For persistence on surfaces, a three-way analysis of variance (ANOVA) followed by a Tukey’s multiple comparisons test was used to reveal interactions between temperature, time and surface type and between temperature, time and initial RNA load on blueberries stored for up to 16 days. Differences in % RNA detected were considered significant at *p* < 0.05.

## Results

### Persistence of Inactivated HAV in Suspension

**FIGURE 1:**
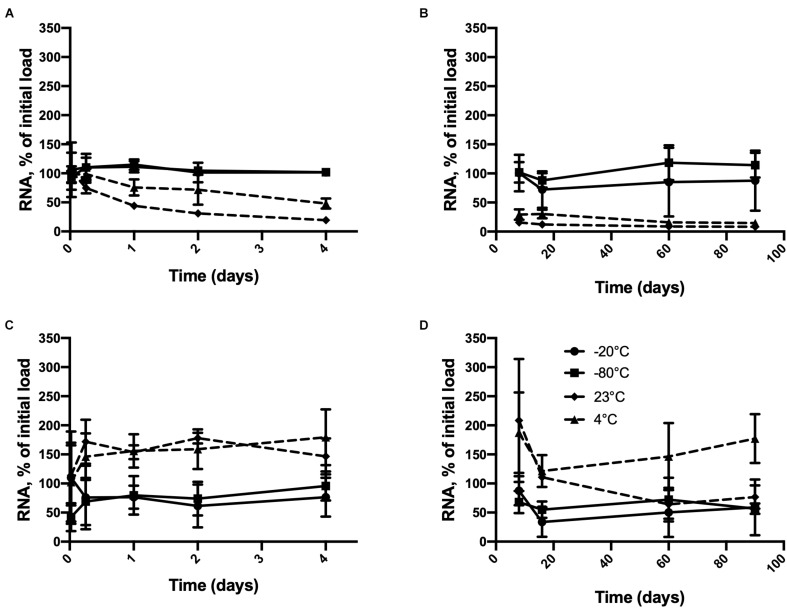
Persistence of inactivated HAV in water (molecular biology grade) initially at 2.5 × 10^6^ genome copies per 100 μL from **(A)** 30 min to 4 days and **(B)** 8 days to 90 days and at 2.5 × 10^4^ genome copies per 100 μL from **(C)** 30 min to 4 days and **(D)** 8 days to 90 days at –80°C (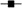),–20°C (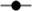), 4° C (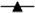), and 23°C (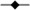). Plotted values are means of 3 repetitions ± standard deviation.

As shown in Figure 1, inactivated HAV remained detectable in pure water stored at all four temperatures for at least 90 days when the initial load was 2.5 × 10^6^ or 2.5 × 10^4^ genome copies. A time × temperature interaction (*p* = 0.0017) was observed at the higher initial RNA concentration (Figures 1A,B). Detectable RNA dropped sharply from this concentration after only 24 h at 23°C compared to −20°C or −80°C. A considerable drop was also observed at 4°C. This difference remained significant for 90 days except on day 16 (*p* = 0.0812). The differences between −20 and −80°C were never significant. Inactivated HAV was nevertheless still detected after 90 days of storage at 23°C (8.67%) and 4°C (14.67%).

Starting from the lower initial load (Figures 1C,D), although no interaction between time and temperature was apparent, each of these factors by itself had a significant effect on detectable RNA (*p* = 0.0094 and <0.0001, respectively). The temperature effect at 60 and 90 days was less consistent than it was in the case of the higher initial load. Inactivated HAV was still detected after 90 days regardless of storage temperature. Starting from a much lower initial load of 250 copies, negative results (RNA not detected) were obtained only at temperatures above freezing, and with one exception, only after 60 days of storage (Table 1). At −20°C and −80°C, inactivated HAV was detected in at least one of three replicates at 60 and 90 days.

**TABLE 1:** Detection of inactivated HAV in water (molecular biology grade) over time at −80°C, −20°C, 4°C, and 23°C starting from an initial load of 2.5 × 10^2^ in 100 μL.

Temperature (°C)		−80	−20	4	23
Time	30 min	3	3	3	3
	6 h	3	2	2	2
	1 day	2	2	3	3
	2 days	2	3	3	**0**
	4 days	2	2	2	2
	8 days	2	3	3	2
	16 days	2	3	1	2
	60 days	2	3	**0**	**0**
	90 days	2	1	2	**0**

*Values are the number of positive RT-qPCR results out of three tests for each experimental condition. Bold was used to present the conditions where RNA was not detected.*

As shown in Table 2, inactivated HAV was detected for up to 90 days at any of the four temperatures when suspended in PBS at intermediate or high copy numbers. At low copy number, its detection was inconsistent in samples stored at low temperatures and doubtful in those stored above freezing temperatures.

**TABLE 2:** Detection of inactivated HAV in PBS stored at −80°C, −20°C, 4°C, and 23°C.

Temperature (°C)		−80			−20			4			23		
Genome copies/sample		250	25,000	2.5E6	250	25,000	2.5E6	250	25,000	2.5E6	250	25,000	2.5E6
Time	0.5 h	1	2	2	2	2	2	2	2	2	**0**	2	2
	6 h	**0**	1	2	1	1	1	**0**	2	2	1	1	2
	1 day	2	2	1	1	2	2	2	2	2	**0**	1	2
	2 days	**0**	2	2	**0**	1	2	**0**	1	2	**0**	2	2
	4 days	**0**	2	2	1	2	2	**0**	1	2	1	2	2
	8 days	1	2	1	**0**	1	2	**0**	**0**	2	**0**	2	2
	16 days	1	1	1	1	1	1	**0**	1	1	**0**	1	1
	60 days	1	1	2	**0**	2	2	**0**	1	2	**0**	**0**	1
	90 days	1	2	2	**0**	1	1	**0**	2	1	**0**	1	2

*Values are the number of positive RT-qPCR results out of two tests for each experimental condition. Sample volume was 100 μL. Bold was used to present the conditions where RNA was not detected.*

### Persistence of Inactivated HAV on Non-porous Surfaces

**FIGURE 2:**
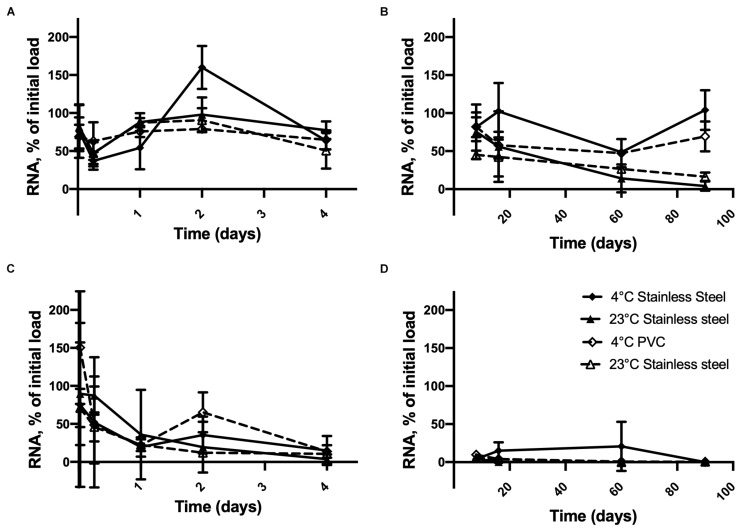
Persistence of inactivated HAV dried on inert material surfaces initially at 2.5 × 10^6^ genome copies per unit (disk, diameter 1 cm) from **(A)** 30 min to 4 days and **(B)** 8 days to 90 days and at 2.5 × 10^4^ genome copies per unit from **(C)** 30 min to 4 days and **(D)** 8 days to 90 days at 4° C on SS (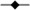), 23°C on SS (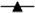), 4° C on PVC (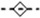), and 23°C on PVC (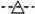). Plotted values are means of 3 repetitions ± standard deviation.

Figures 2A,B shows that inactivated HAV was still detectable on the 90th day of storage on stainless steel or PVC at 4°C when the initial load was 2.5 × 10^6^ copies. At this concentration, time × temperature (*p* < 0.0001), time × surface (*p* = 0.0302), and time × surface × temperature (*p* = 0.0251) interactions were observed. This results in similar inactivated HAV detection regardless of the storage conditions tested, except at 90 days, when differences emerged between 4 and 23°C for both materials. At this point, >69% of the initial inactivated HAV was detected on PVC and stainless steel at 4°C, compared to <17% at 23°C.

When the initial inactivated HAV load dried on the material surfaces was smaller (Figures 2C,D), the amount detected by RT-qPCR dropped to less than 15% within 4 days at all temperatures. In fact, time was the only significant factor (*p* < 0.0001). On stainless steel, inactivated HAV was barely detectable (0.33%) on day 16 at 23°C and not detected at all on day 60 or 90 or on day 90 at 4°C. It was detected on PVC at 4°C (0.33%) and at 23°C (0.50%) on day 90. Experiments with 250 genome copies per surface unit gave highly inconsistent results (not shown).

### Persistence of Inactivated HAV on Blueberries

**FIGURE 3:**
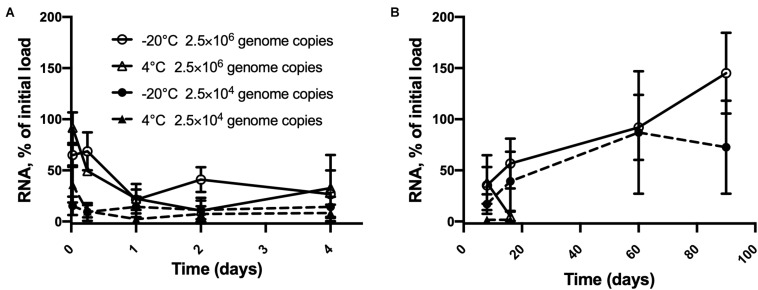
Persistence of inactivated HAV dried on blueberries at two initial loads from **(A)** 30 min to 4 days and **(B)** 8 days to 90 days. 2.5 × 10^6^ genome copies per blueberry at –20°C (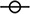) and 4° C (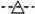) and 2.5 × 10^4^ genome copies per blueberry at –20°C (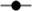) and 4° C (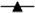) Plotted values are means of 3 repetitions ± standard deviation.

As shown in Figure 3, inactivated HAV at high copy numbers on blueberries remained fully detectable up to 90 days at −20°C. At this temperature, time (*p* < 0.0001), and concentration (*p* = 0.0001), each had a significant effect on detection but did not interact statistically. From 30 min to 16 days, time and concentration interacted (*p* = 0.0023) as did time and temperature (*p* = 0.0002). The 22% drop from 2.5 × 10^6^ within 24 h at 4°C is significant (*p* = 0.0002), as is the subsequent drop to only 5% of the initial load by day 16. Stored at −20°C, the same initial load gave erratic assay results, but these did not vary significantly from one time point to the next, notwithstanding the apparent outlier at 90 days. In the case of 25,000 copies per berry, the overall change was smaller, since even the first assay detected less than 50% of this number. Detection was down to 2% on days 8 and 16. Temperature made little difference over this time interval. Although detection peaked at 87% on day 60 at −20°C, this value was not significantly different from that measured at the 30 min point.

## Discussion

In this study, we measured the persistence of inactivated HAV under different conditions of temperature, initial load, medium, and surface in order to investigate the potential influence of non-infectious viral particles on the assessment of risks of enteric illness spreading via foods and food-handling surfaces. Our results showed that at medium-to-high copy numbers of RNA, inactivated HAV associated with non-infectious virions could persist for long periods in suspension or ice, on stainless steel or PVC and on blueberries. As mentioned elsewhere, few molecular detection methods discriminate between genetic material and truly infectious viruses detected in foods. However, reference methods such as ISO 15216-1:2017 for the detection of enteric viruses in food matrices (Anonymous, 2017) and the United States standard from the *Bacteriological Analytical Manual* on the detection of HAV in foods (Williams-Woods et al., 2017) and methods in development such as ddPCR, next-generation sequencing (Coudray-Meunier et al., 2015; Bartsch et al., 2018), or direct lysis (Sun et al., 2019; Rajiuddin et al., 2020) are all based entirely on genome detection. The present study shows how test results based solely on RNA detection could be misleading and that overreliance on them could be prejudicial to businesses under investigation with regard to a suspected outbreak of viral gastroenteritis.

It is generally agreed that mRNA is relatively unstable, with a half-life varying from a few minutes (Bernstein et al., 2002; Selinger et al., 2003) to several hours (Krowczynska et al., 1985; Shyu et al., 1989; Sharova et al., 2009) depending on the species. The persistence of detectable viral RNA in the environment has received relatively little attention. It has been shown that the murine norovirus (MNV-1) genome can persist on stainless steel for up to 24 days at room temperature (Escudero et al., 2012). In the same study, purified human NoV RNA (GI or Norwalk virus and GII or Snow mountain virus) could be detected for up to 7 days. Under similar conditions, purified Norwalk virus RNA appeared to remain detectable for 24 h (D’Souza et al., 2006). These findings are consistent with our results, which suggest that RNA breaks down quickly on stainless steel at room temperature. However, our study differs from those of Escudero et al. (2012) and D’Souza et al. (2006) since we used heat-inactivated virions rather than purified RNA in order to include damaged capsids in our conditions. Furthermore, we chose molecular-biology-grade water rather than PBS to elute inactivated HAV and thereby avoided possible inhibition of the RT-qPCR reaction by the buffer. Our results nevertheless show that under conditions routinely encountered in the food distribution and consumption chain, inactivated HAV can persist for periods much longer than mRNA.

Our results show that inactivated HAV can remain detectable at levels that vary relatively little over time when suspended at copy numbers ranging from 2.5 × 10^4^ to 2.5 × 10^6^ in sterile molecular-biology-grade water stored at −80°C or −20°C. These temperatures are recommended in standard methods of conserving viral genomes over long periods (Anonymous, 2017; Williams-Woods et al., 2017). We compared them to refrigeration (4°C) and room temperature (23°C) using the same copy numbers. These initial loads can remain 8% detectable (a modest reduction of 1.1 log) after 90 days at 23°C. The dynamics appear to vary considerably with copy number, and at least 75% of the initial inactivated HAV load may be detected at the 90-day point even when starting at 25,000 genome copies per sample. However, the error bars were large, and further analyses are required to corroborate this hypothesis. Single-stranded RNA viruses have been shown to persist for up to 18 days in autoclaved Milli Q water and wastewater stored at 20°C and for 28 days in filtered seawater stored at room temperature (Tsai et al., 1995; Limsawat and Ohgaki, 1997), possibly due to the presence of microorganisms (Tsai et al., 1995; Limsawat and Ohgaki, 1997). Our results show that in sterile, ribonuclease-free water, viral RNA from inactivated HAV can persist at least three times longer at 4°C or room temperature.

Inactivated HAV was detectable in PBS but quantification was difficult at high copy numbers and not possible at low or intermediate numbers, due likely to inhibition of RT-qPCR reactions. The *Ct* values of the test samples and the positive controls were highly variable. Although it is known that salts can inhibit the PCR reaction (Rossen et al., 1992; Schrader et al., 2012), RNA persistence could still be evaluated qualitatively in PBS since several samples were positive for 90 days.

Although inactivated HAV also degraded quickly on blueberries at 4°C, it could still be detected after 16 days. It has been shown that MNV-1 RNA and GII human norovirus remain detectable for at least 14 days on lettuce kept at 4°C or at room temperature (Escudero et al., 2012). Since the persistence of virion infectiousness is affected by environmental conditions (Bae et al., 2014; Leblanc et al., 2019) as well as intrinsic characteristics of the virus species (Cannon et al., 2006; Lamhoujeb et al., 2008; Provost et al., 2011), it is not surprising that the initial RNA copy number, the storage temperature, and the type of surface have a strong influence on the persistence of detectable RNA associated with inactivated virions. The liquid used to elute the virus from the test materials and fruit could also have affected the results, since inactivated HAV detected in PBS dropped over time at room temperature, 4°C and −20°C starting from lower copy numbers, compared to results obtained when using water. Persistence of detectable RNA under these conditions also seems to be influenced by viral species, strain, and the composition of the contaminating medium (D’Souza et al., 2006; Escudero et al., 2012).

On blueberries stored at −20°C, inactivated HAV could be detected at levels close to the initial load for up to 90 days. This needs to be taken into consideration, given that frozen berries can be stored for long periods by distributors and subsequently by consumers (Hutin et al., 1999; Collier et al., 2014; Müller et al., 2015). For this reason, frozen berries have been implicated on numerous occasions in outbreaks of foodborne viral gastroenteritis and are closely monitored by government agencies (reviewed by Tavoschi et al., 2015; Nasheri et al., 2019). Regardless of the variety of treatments used to reduce the infectious viral load on the surfaces of frozen produce, our results suggest that residual inactivated virus may persist for several months at low temperatures, and its detection could be interpreted as the presence of infectious virus.

The standard deviation of a few of the results obtained for blueberries needs explaining, especially in the case of storage at −20°C. Berries are known for their content of phenolic substances and are highly valued by consumers for their antioxidant properties (Cooke et al., 2005; Gilbert et al., 2014; Olas, 2018). However, some of these compounds have been reported to inhibit PCR reactions (John, 1992; Wei et al., 2008; Schrader et al., 2012). In fact, this is a recurring obstacle to the detection of enteric viruses on fruits, and various methods of reducing the amount of inhibitory substance in samples for analysis have been investigated (Bartsch et al., 2016b; Sun et al., 2019). In the present study, no purification step was carried out on the samples. In addition, slow freezing at −20°C followed by thawing could have exacerbated the release of phenolic compounds (de Ancos et al., 2000; Reque et al., 2014). Reque et al. (2014) also showed an increase in the activity of anthocyanins in frozen blueberries after 3 months of storage followed by a decrease during the subsequent 3 months. Lastly, the presence of pectin found in blueberries may also inhibit RT-qPCR (Suther and Moore, 2019). These findings are consistent with the significant variations seen in our detections of HAV RNA at 60 and 90 days of storage.

As our results suggest, the persistence of viral RNA on surfaces, on fruits, and in suspension could contribute considerably to positive results of tests intended to detect infectious viruses throughout the food chain. In order to prevent misinterpretation of the real risk to consumers, the detection method should include some means of differentiating between infectious and non-infectious virions. This subject has been examined widely (Nuanualsuwan and Cliver, 2002; Baert et al., 2008; Lamhoujeb et al., 2008; Parshionikar et al., 2010; Sánchez et al., 2012; Moreno et al., 2015; Zhang et al., 2019; Escudero-Abarca et al., 2020). Methods in development have been described in the literature, but none has been standardized to date. Although the efficacy of previous enzymatic treatments was debatable (Nuanualsuwan and Cliver, 2002, 2003; Baert et al., 2008; Lamhoujeb et al., 2008), several new strategies are now available. Pretreatment with proteinase K to degrade already damaged capsids followed by ribonuclease to cleave the viral RNA released was an obvious first approach (Nuanualsuwan and Cliver, 2002). Infectious HAV, poliovirus1, and feline calicivirus can be selected by such means if the viral particles are previously inactivated by UV, sodium hypochlorite, or heat. However, in the case of heat inactivation of MNV-1, the correlation between the number of infectious particles and the number of genome copies was poor (Baert et al., 2008). Detection of heat-inactivated NoV genetic matter could be reduced substantially by this treatment in conjunction with NASBA (Lamhoujeb et al., 2008). In a study of persistence on stainless steel at room temperature, treatment with ribonuclease alone was found not to allow detection of infectious MNV-1 particles only, since this enzyme could not degrade viral RNA inside intact non-infectious particles (Leblanc et al., 2019). Combining RT-qPCR with a non-enzymatic pretreatment such as propidium monoazide has been proposed to differentiate between infectious and non-infectious viral particles (Sánchez et al., 2012; Leifels et al., 2015; Quijada et al., 2016). This approach has been found more effective than RNase treatment for detecting infectious HAV in conjunction with thermal inactivation (Sánchez et al., 2012). Although these pretreatments show potential for allowing the detection of infectious virions only, they are limited by the inactivation of the particles of interest by damage to the capsid (Nuanualsuwan and Cliver, 2003; Zhang et al., 2019).

By performing the RT-qPCR analyses on the day that the inactivated HAV was recovered from solutions or eluted from food contact surfaces and blueberries, the bias potentially introduced by sample storage and thawing was eliminated. However, there could have been inter plate differences between our experimental replicates over the 90-day period. Therefore, we implemented several procedural controls on each assay plate and presented the results in light of those controls, which we believe would have alerted us to deviations in technical consistency. We are aware that heat-inactivating treatment could enhance RNA stabilization with potential capsid ribonucleoproteins, a phenomenon previously described when heating poliovirus at 72°C, 2 min (Knight et al., 2013). However, additional data are required to further establish this hypothesis under our conditions.

This study provides a general idea of the extent to which enteropathogenic viruses introduced by sporadic or systemic contamination might persist as detectable RNA at different points in the industrial food chain. RNA associated with inactivated HAV does persist considerably over time on non-porous surfaces and on foods at a wide range of temperatures. The persistence at temperatures above freezing, although lesser, is cause for concern. These results show that molecular methods of detecting viruses throughout the food production and distribution chain, especially in the context of investigating outbreaks and possibly ordering onerous product recalls, need to include suitable means of distinguishing between non-infectious residues of viruses and truly infectious particles. This could take the form of a sample pre-treatment protocol.

## Data Availability Statement

The raw data supporting the conclusions of this article will be made available by the authors, without undue reservation.

## Author Contributions

MT-F designed the study, performed the experiments and analyses, and wrote the manuscript. EJ provided technical support and assisted the writing of the manuscript. JJ supervised every step of the study. All authors contributed to the article and approved the submitted version.

## Conflict of Interest

The authors declare that the research was conducted in the absence of any commercial or financial relationships that could be construed as a potential conflict of interest.
